# Disorganized cortical thickness covariance network in major depressive disorder implicated by aberrant hubs in large-scale networks

**DOI:** 10.1038/srep27964

**Published:** 2016-06-15

**Authors:** Tao Wang, Kangcheng Wang, Hang Qu, Jingjing Zhou, Qi Li, Zhou Deng, Xue Du, Fajin Lv, Gaoping Ren, Jing Guo, Jiang Qiu, Peng Xie

**Affiliations:** 1 Department of Neurology, The First Affiliated Hospital of Chongqing Medical University, Chongqing, China; 2 Chongqing Key Laboratory of Neurobiology, Chongqing, China; 3 Institute of Neuroscience and the Collaborative Innovation Center for Brain Science, Chongqing Medical University, Chongqing, China; 4 School of Psychology, Southwest University, Chongqing, China; 5 Key Laboratory of Cognition and Personality, Ministry of Education, Southwest University, Chongqing 400715, China; 6 Department of Radiology, The First Affiliated Hospital of Chongqing Medical University, Chongqing, China

## Abstract

Major depressive disorder is associated with abnormal anatomical and functional connectivity, yet alterations in whole cortical thickness topology remain unknown. Here, we examined cortical thickness in medication-free adult depression patients (*n* = 76) and matched healthy controls (*n* = 116). Inter-regional correlation was performed to construct brain networks. By applying graph theory analysis, global (i.e., small-worldness) and regional (centrality) topology was compared between major depressive disorder patients and healthy controls. We found that in depression patients, topological organization of the cortical thickness network shifted towards randomness, and lower small-worldness was driven by a decreased clustering coefficient. Consistently, altered nodal centrality was identified in the isthmus of the cingulate cortex, insula, supra-marginal gyrus, middle temporal gyrus and inferior parietal gyrus, all of which are components within the default mode, salience and central executive networks. Disrupted nodes anchored in the default mode and executive networks were associated with depression severity. The brain systems involved sustain core symptoms in depression and implicate a structural basis for depression. Our results highlight the possibility that developmental and genetic factors are crucial to understand the neuropathology of depression.

Major depressive disorder (MDD) is one of the psychiatric disorders with the highest prevalence, and has received much attention from the public and researchers. The key manifestations of depression are disturbed mood, reward and cognitive profiles^1^,^2^. With the advance of magnetic resonance imaging (MRI) techniques, the neural correlates of depression have attracted great interest. Consequently, studies have identified brain regions with structural abnormalities. Accordingly, grey matter shrinkage has been confirmed by imaging studies^3^. A constellation of cortical thickness differences are commonly reported in the cingulate cortex, dorsal prefrontal cortex, orbitofrontal cortex and temporal lobe^4^,^5^,^6^. These grey matter abnormalities are relevant to the depressive state or vulnerability.

Interestingly, a previous study found an increased correlation among regions with cortical thickness changes in MDD^6^. This indicates that neuropsychopathology in MDD cannot simply be attributed to focal grey matter loss, and the underlying neuronal coordination in MDD should also be taken into account. Inter-regional grey matter covariance is dominated by common underlying factors such as neurotrophic factors for direct anatomical connections^7^, genetics^8^, developmental processes^9^ and functional specificity^10^. Grey matter covariance can be measured by various metrics (e.g., thickness, area and volume). Empirical studies have shown that cortical thickness and area are genetically independent measurements^11^. This independence also applies to their topological organization^12^. Since grey matter volume is product of thickness and area, it would be more biologically interpretable to separate cortical area and thickness in studies. These findings also indicate that it is appropriate to use thickness as an endo-phenotype for investigating neuropsychiatric disorders.

Disrupted brain connectivity in psychiatric disorders commonly involves large-scale networks, especially the default mode network (DMN)^13^. Conventional approaches focus on only a single network component and do not consider the interactions among the whole brain. This reductionist view is useful for functional localization, but difficult for elaborating on functional integration. Brain regions and functional networks may be segregated by specificity, yet the whole active brain is wired by integration. Given that depressive disorder has heterogeneous manifestations in affective, cognitive and vegetative nervous domains, it is natural to assume that these symptoms arise from discrete brain systems^1^,^2^, and that interactions among brain systems might contribute to its neuropsychopathology^14^.

Network science has provided powerful analytical tools to examine the complex interactions of cerebral organization. Graph model analysis uses a straightforward implication of how information is efficiently transmitted through networks. Brain networks are constructed by segmenting the brain into discrete regions (denoted as nodes), and then the coupling relationship between two nodes (denoted as the edge) is determined. Many quantitative scalars have been proposed to demonstrate how efficiently information is transmitted through the whole network or passes through a single node. This approach has quantitatively revealed a “small-world” human brain^15^. The small-world scalar is defined as the ratio of the speed of information transmitted among neighbouring nodes^16^,^17^ (i.e., clustering or local efficiency, which measures segregation) to remote nodes (i.e., path length or global efficiency, which measures integration). Typically, if a shortcut exists between remote nodes, this network is labelled as a small-world network. Besides the global metric, the role of one component in the network may also be of interest. Nodes that are critical for the maintenance of efficient information flow are nominated as hubs. The disrupted connectome and brain hub failure may be key to understanding psychiatric disorders^14^,^18^. For functional and anatomical MRI, networks are intuitively constructed on an individual level. Initially, the node is mapped onto subdivisions of the brain atlas by cytoarchitecture or another factor. For functional connectivity (e.g resting-state fMRI), the connections are defined as synchronization of segregated, distributed neurophysiological activations (or statistical temporal dependency); anatomical connections are white-matter bundles reconstructed by diffusion MRI. An alternative approach is to use covariance of the morphological metric across subjects as connections^19^. The structural covariance network (SCN) most likely reflects coordination of cerebral maturation process^20^. Studies have shown that thickness covariance networks also preserve small-world network architecture as well as functional/anatomical networks. Besides the developmental aspect, the topology of SCN is also partially influenced by anatomical connections^21^, or co-activity especially those regions implicated in sensory-motor, language system^10^.

It is important to address whether brain pathology in depression exhibit a specific pattern at network level. In this study, instead of assessing regional grey matter abnormalities, we sought to determine disrupted cortical covariance patterns from a large sample of depression cases. We adopted graph theory analysis to highlight disrupted cortical architecture underpinning the neuropathological process. Based on previous adult depression studies that have reported aberrant small-world and hubs topology in functional and structural network^22^,^23^,^24^, we hypothesize that: 1) SCN of cortical thickness exhibits disrupted global topology; 2) hub location in the depressed brain should be similar to previous findings, including frontal and cingulate midline structures; and 3) disrupted nodal topology is relevant to the psychopathology of depressive disorder.

## Results

### Demographic features of participants

Demographic data from both groups were analysed in R (version 3.1.0, http://cran.r-project.org/). Sixteen of 76 patients were first episode, while the remaining 60 patients had reported one or more previous episodes. However, we did not record the number of episodes as no valid medical records were found. Details are listed in Table 1. The demographic features did not differ between HC and MDD group, as well as between MDD subgroups.

### Loss of small-world was driven by reduced clustering

**Figure 1:**
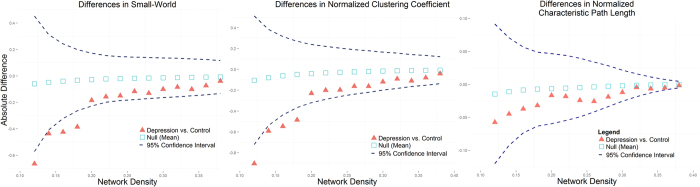
Differences in small-worldness (σ, left panel), clustering coefficients (γ, middle panel) and characteristic path length (λ, right panel) across connection densities of 0.12–0.4. The red triangle denotes a difference between MDD and HC groups, the black square denotes a difference between the permutation null network, while blue dash lines denote 95% confidence intervals.

In the MDD group, predominantly reduced covariance connectivity (*n* = 44 pairs reduced connection, *n* = 20 elevated connection; total of 228 connections) was detected (Supplemental Material, Fig. S1). In both healthy and depressed groups, thickness SCN showed a small-world topology (σ >> 1 in all density ranges). Lower σ was detected in the patient group at a sparsity range between 0.12 and 0.18 (all *p* < 0.05). Lower σ was mainly driven by a lower normalized clustering coefficient (γ, all *p* < 0.05) at the same sparsity range because normalized characteristic path length (λ) did not differ (all *p* > 0.1, Fig. 1). We compared these sparsity-dependent measures by AUC and FDA analyses, yielding identical results of lowered σ (*p* = 0.018) and lowered γ (*p* = 0.035), but not λ (*p* = 0.362). Deviated global efficiency was not observed between groups (*p* = 0.473) by AUC/ FDA analysis, although there was a trend towards lower local efficiency (*p* = 0.064). Validation analysis also found a lower small-world (*p* = 0.006) in the MDD group, which was driven by lower γ (*p* = 0.004) (Supplemental Material, Fig. S2 and Table S1).

**Figure 2:**
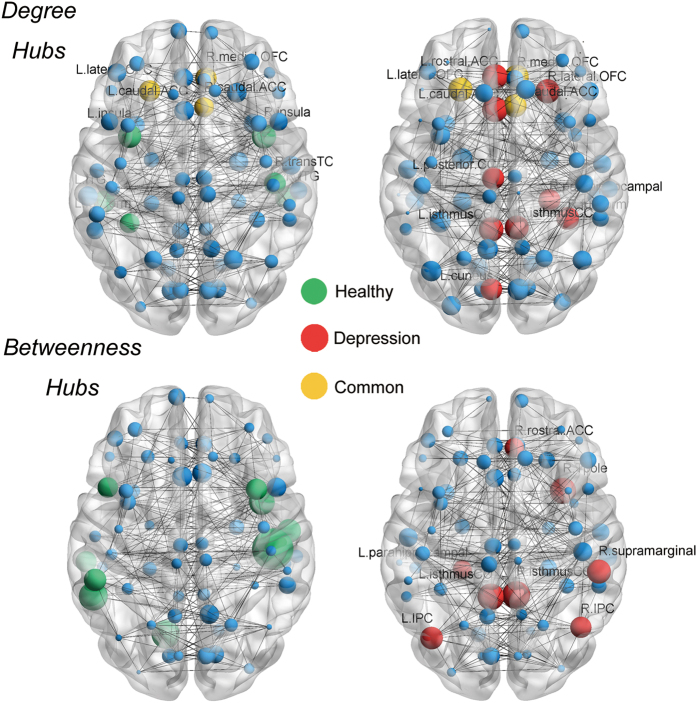
Constructed network and hub distribution. All nodes are visualized as spheres and size is weighted by individual normalized degree/BC. Non-hubs are coloured in blue, green spheres are hubs specific to HCs, red spheres are hubs specific to MDD patients, and yellow spheres are hubs in common. Degree hubs specific to HCs (green): fusiform, inferior temporal gyrus and insula in the left hemisphere; and inferior temporal gyrus, transverse temporal gyrus and insula in the right hemisphere. Degree hubs specific to MDD patients (red) include: caudal anterior cingulate gyrus, cuneus gyrus, isthmus of the cingulate cortex, posterior cingulate cortex and rostral anterior cingulate cortex in the left hemisphere; and fusiform, isthmus of the cingulate cortex, lateral orbitofrontal gyrus and parahippocampal gyrus in the right hemisphere. Degree hubs in common (yellow): lateral orbitofrontal gyrus in the left hemisphere; and caudal anterior cingulate cortex, and medial orbitofrontal gyrus in the right hemisphere. Completely non-overlapping betweenness hubs were identified by comparing HCs with MDD patients. Betweenness hubs specific to HCs: banks of the superior temporal sulcus, lingual gyrus, middle temporal gyrus, pars opecularis and supra-marginal gyrus in the left hemisphere; and caudal middle frontal gyrus, superior temporal gyrus, transverse temporal gyrus and insula in the right hemisphere. Betweenness hubs specific to MDD patients: inferior parietal gyrus, isthmus of the cingulate cortex and parahippocampal gyrus in the left hemisphere; and isthmus of the cingulate cortex, rostral anterior cingulate cortex, supra-marginal gyrus and temporal pole in the right hemisphere.

Nodal centrality was first normalized by overall mean. Hubs were defined as one standard deviation above the mean of centrality. Hub number and spatial distribution displayed divergence between groups. Degree hubs were distributed in approximate symmetry in bilateral hemispheres. In HCs, there was a total of nine degree hubs involving bilateral orbitofrontal, bilateral inferior temporal, bilateral insula, left fusiform, right caudal anterior cingulate and right transverse temporal regions. Twelve MDD hubs were concentrated in midline structures, including six cingulate subdivisions, the orbitofrontal gyrus and the temporal lobe region. Only three hubs in the MDD group overlapped with HCs (Fig. 2, upper panel). We identified completely non-overlapping hubs by using betweenness (BC) to compare MDD patients (*n* = 8) and HCs (*n* = 9) (Fig. 2, lower panel).

### Disrupted nodal centrality and its association with depression severity

**Figure 3:**
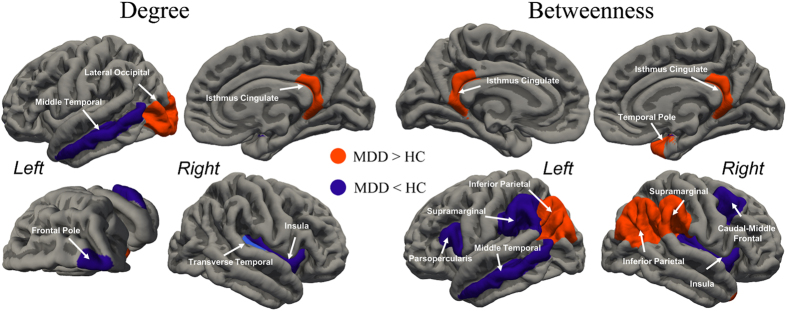
Differences in regional centrality between MDD patients and HCs (*p* < 0.05 after AUC analysis and FDR corrected, 5000 permutations). Regions showing decreased centrality are coloured in purple, while regions showing increased centrality are coloured in orange.

Alterations in nodal centrality were mainly identified in hub regions. The MDD group showed a greater degree of centrality in the left occipital and right isthmus of the cingulate, and with greater BC in the bilateral inferior parietal, bilateral isthmus of the cingulate, right supra-marginal and right temporal pole (*p* < 0.05, FDR corrected). In contrast, the MDD group had a lower degree of centrality in the left middle temporal, right frontal pole, right transverse temporal and right insula cortex, with lower BC in the middle temporal, pars opercularis (i.e ventral lateral prefrontal/ VLPFC) and supra-marginal of the left hemisphere, and caudal middle frontal, transverse temporal and insula cortex of the right hemisphere (*p* < 0.05, FDR corrected, Fig. 3). Hubs predominantly related to the MDD group observed increasing centrality, while decreased centrality nodes were mostly hubs in the HC group (Table 2). For MDD, nodal efficiency was lower in left rostral middle frontal (i.e dorsal lateral prefrontal/ DLPFC), right entorhinal and right temporal pole; while higher efficiency was discovered in right cuneus and caudal anterior cingulate (Table 2). Validation analysis showed that regions with altered nodal centrality did not completely overlap with our results from the 68 × 68 network (Supplemental Material, Fig. S4 and Table S2).

**Figure 4:**
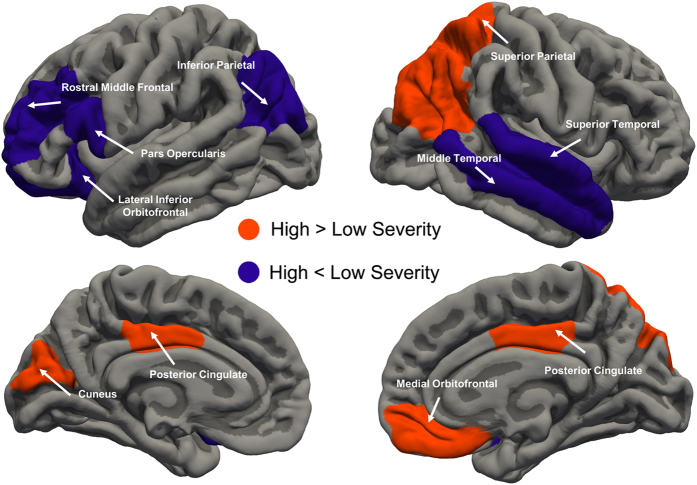
Changes in nodal centrality (combination of degree, betweenness and efficiency) associated with depression severity. Compared with patients with less illness severity, greater severity is associated with increased centrality in the bilateral mid-posterior cingulate cortex, right superior/inferior parietal cortex and right medial orbitofrontal (orange regions). Lower centrality in the left inferior parietal and lateral orbitofrontal cortex is associated with less severity (purple regions).

Within the MDD patient group, greater severity did not indicate alteration global topology across all network density (σ-pval = 0.42, γ-pval = 0.566 and λ-pval = 0.723). Higher centrality was identified in bilateral mid-posterior cingulate cortex, right superior/inferior parietal cortex and right medial orbitofrontal cortex was associated with greater severity. Lower centrality was observed in left inferior parietal, lateral orbitofrontal and right superior temporal cortex in high severity group (Fig. 4, Table 3). We observed that high severity was associated with lower nodal efficiency in left rostral middle frontal and pars opercularis cortex, higher efficiency in left cuneus and right middle temporal cortex (Table 3).

## Discussion

The main contribution of our study is the demonstration of disrupted cortical thickness organization in medication-free major depression patients. Generally widespread reduced connectivity was observed in MDD, especially connections within and between frontal, temporal and limbic areas. Global architecture of cortical thickness in MDD patients shifted toward randomness, which was driven by decreased clustering. Nodes with abnormal centrality included areas at the core of the DMN, that is salience network (SN) and central executive network (CEN). Illness severity was associated with disrupted centrality nodes involved in executive, self-referential and emotional processes (i.e. parietal, orbitofrontal and cingulate regions). This suggests that altered large-scale network connectivity contributes to the underlying psychopathy. Our results further suggest that developmental and genetic factors might also be crucial to understand the process of pathogenesis.

### Interpretation of Disrupted Cortical Thickness Topology in MDD

Recently, study revealed that the SCN reflect the synchronized cerebral maturation process during adolescent^20^; developmental trajectory of cortical thickness network also conserved small-world architecture. High clustering in small-world architecture facilitates functional specificity (segregation) and gives rise to several large-scale functional networks, e.g. default mode, executive-control and salience networks^25^. However segregation in SCN does not necessarily reflect local processing capacity. It is on debate whether global topology is disrupted in MDD. Resting-state functional networks reveal decreased global clustering, altered path length and altered global efficiency^26^,^27^. Using a subset of our study sample, Luo and colleagues identified altered global topology in a functional network^27^, which is strikingly similar to our thickness network presented here. It should be note that in healthy population, approximate 60% of thickness correlation are similar to functional network, which are possibly result of neuronal synchronization induces synaptogenesis^28^. In MDD patients, the less clustered and randomized configuration of cortical thickness network may undermine large-scale network and corresponding functional capacity. We observed widespread loss of grey matter coupling (Fig. S1, Table 2) mainly targeted on frontal, temporal and para-limbic regions, which collectively cause the reduced clustering. Studies have also confirmed disrupted white matter integrity in frontal-subcortical, frontal-limbic and inter-cortices fibres^29^,^30^. One anatomical connectome analysis revealed a longer path length in MDD^24^ and two hypo-connected subnetwork, while others found no alteration^31^. Despite potential clinical heterogeneity, this discrepancy may be interpreted as the uniqueness not shared with anatomical network^21^.

Although volume-constructed connectome^23^ had strikingly similar results to ours, at least a proportion of the altered topology may root in clinical heterogeneity (medication and comorbidities). Study reported greater clustering and lower global efficiency in late-life depression^32^ are contrary to adult depression, possibly implying distinct underlying neuropathology of illness due to age. These indirect evidences collectively demonstrate the unique role of grey matter covariance as complementary to functional and anatomical studies. In the future, multi-modal and genomic imaging research should be performed to reveal the contribution of particular genetic influences to neural plasticity, as well as gene-environment interactions^33^. The current findings shed light on the neuropathology of MDD on two folds: first, disrupted function network was embedded in abnormal organized SCN. Second, the aberrant topology in SCN was partially shaped during a critical period of brain development. Lower segregation is a possible quantitative illustration of disconnection syndrome in MDD^34^.

Childhood maltreatment (or early life stress, ELS) may contribute to depression risk and related neurobiological consequences. SCN with childhood maltreatment produces altered centrality in anterior cingulate, temporal pole and anterior insula^35^ which is similar to our finding. RS-fMRI revealed that women with ELS have decreased centrality in prefrontal cortices, while only in ELS with history/current depression exhibited amygdala and caudate centrality^36^. Another study found more seriously decreased thalamus degree in depression with ELS, and negatively correlated with maltreatment level^37^. We speculate ELS in depression produces more complicated neurobiological impairment in sub-cortical regions. Indeed it has reported that topology of striatum is associated with more depressive episode^26^; and topology of hippocampus negatively correlated with severity and duration of illness, caudate nucleus positively correlated with severity^22^. Gene-environment interactions have significant effects in predicting grey matter covariance in depression. Several genotypes which may influence serotonin^37^, dopamine^38^ and brain-derived neurotrophic factor^38^ have a profound impact on covariance and functional connectivity strength, of which are particularly focused in MDD. High DMN connectivity is supposed to be neural signature of depressive rumination^39^, meanwhile increased DMN connectivity is associated with high family risk^40^. Taken together, combination of environmental and genetic factors may contribute to the less optimized cerebral thickness topology in MDD.

Hubs in depression group may explain sustained depressive symptoms in patients. Increased topological importance in cingulate cortices may be associate with abnormal affective process and its regulation in MDD. The subgenual cingulate cortex has been identified morphological and functional abnormalities^41^ in extensive literature. The caudal anterior cingulate of MDD patient is hyperactivated in outcome evaluation task and emotional regulation task^42^, and hypoactivated in the emotional face discrimination task^43^. The isthmus of the cingulate cortex is at the core of the DMN, which is profoundly involved in maladaptive rumination in depression^39^,^44^. Increased DMN functional connectivity is also found in subjects with high depression risk^40^. Activity in the DMN is closely correlated to self-oriented rumination, the frequency of rumination is highly related to negative emotion in healthy people^45^. Hyperactivity of DMN can be diminished by antidepressant treatment^46^,^47^. Together, disturbed activity and structure in cingulate subdivisions is highly associated with several core aspects of MDD. Identified disrupted occipital node may be due to visual processing biases; several studies reported that grey matter alterations in MDD are associate with co-occurring anxiety^48^,^49^.

The small-world model does not characterize hubs, however hubs may emerge from a preferential-attachment-like process. The human brain connectome represents an intermediate state between small-world and scale-free networks. Most studies implicate a truncated power-law distribution in the connectome^7^, which brings up not huge hubs, but several hubs. This cerebral architecture shows resilience to hub failure^15^, while on the contrary, disrupted global topology in MDD is consequentially associated with alteration of hubs. Astonishingly, a large proportion of hubs re-allocated into para-limbic areas (Cingulate cortices, Fig. 2). Overall, lowered centrality and increased trivial hubs may contribute to disrupted global topology. In addition to topological abnormalities, these hub regions are also widely linked to regional abnormalities in previous studies^3^,^43^. Indeed, the results of the univariate approach converge with the connectome approach to reveal a common biological basis for depression. Emergence of a human brain connectome might be the result of a trade-off between physiological cost and topological efficiency^50^. Metabolic demand of hubs is relatively higher than non-hubs^51^, which shows that hubs are vulnerable to a bio-pathological deficit. Meta-analysis and simulation indicate that hubs are especially vulnerable in neuropsychiatric disorders^18^. Another explanation for the hub-targeted pattern is simply their high topological value, causing peripheral lesions to propagate and to concentrate in hubs.

### A Network Model of Depression Implicated by Aberrant Hubs in Large-scale networks

Nodes detected with altered centrality are hubs in either group. Consistently we identified group differences in centrality in the bilateral isthmus of the cingulate, middle temporal gyrus, insula, inferior parietal lobule and middle/inferior frontal gyrus (Fig. 3, Table 2). The collective disrupted nodes are pivots of three main large-scale networks: default mode, salience and central executive networks. Disrupted hub centrality in DMN, SN and CEN nodes has also been shown in previous network studies^22^,^23^,^27^,^31^,^32^, which imply the inter-triple-network disruption hypothesis in MDD^52^. Compelling evidence from a causal model has shown that the right insula is responsible for switching between CEN and DMN^53^,^54^. The right insula is implicated in saliency detection during perceptive, affective and interoceptive stimuli^55^; therefore, the insula is a critical node that mediates interactions between goal-directed and automatic processes. In other words, the right insula in SN is crucial for manipulation of information flow between external and internal mental states. As a corollary of its neuropsychological significance, diminished activation in MDD patients has been shown during an interoceptive task, which was accompanied by hyper-connectivity to limbic regions^56^. Despite putative psychological functions ascribed to these systems, disturbed dynamic interactions among the triple-network shed light on mechanisms of MDD. Evidence has shown decreased connectivity between DMN and CEN in MDD patients, and this disrupted connectivity is predicted by right insula activity^57^. Hamilton *et al*. reported disrupted DMN–CEN interaction effects located in the right insula, and a double dissociation of insula fluctuation within DMN and CEN^44^. Clearly, the SN in depressed people is incapable of properly filtering information. More importantly, failure of SN to assign a top-down regulation system to processing information leads to sustained negative thoughts. Malfunction of CEN is likely to be responsible given its attenuated intrinsic connectivity^58^. Consequently, in MDD patients, their affective system receives and processes more negative stimuli. Depressive maladaptive rumination is dominated and internalized in DMN, and fails to deploy ‘top-down’ regulation. This disrupted neural substrate may account for several aspects of manifestation in MDD, namely pervasive subjective negative feelings, difficulty in disengaging from depressive rumination, and subsequent failure in ‘top-down’ emotional regulation. Altogether, we heuristically propose that MDD can be characterized by malfunction in the switching process between external (undermined top-down regulation in CEN) and internal (negative self-oriented thoughts in DMN) states mediated by disrupted capacity of salience processing in the insula.

Nodes associated with executive, self-referential and emotional processing distinguish patients with greater illness severity (Fig. 4, Table 3). The middle frontal cortex and inferior parietal cortex consistently displays decreased local topology in MDD versus HCs, and in less-more severity comparisons. In clinic, stimulation on rostral middle frontal is a novel and efficacious treatment to MDD^59^. Cortical thinning in these regions may mediate family risk and executive function in MDD^60^. We found increased centrality of the left inferior parietal area in MDD, which was decreased in more severe patients. This pattern may indicate a different pathogenesis or lateralization in MDD. Disrupted connectivity anchored in the lateral orbitofrontal–cingulate system has long been considered to be related to valence processing in MDD^61^. The medial orbitofrontal cortex (i.e ventromedial prefrontal cortex) is a DMN subcomponent specialized for self-referential process, its hyper-connectivity may indicate residual symptoms and future relapse after antidepressant treatment^46^. We did not detect aberrant global topology within MDD group, several reasons can account for the phenomenon. First, changes in local topological measurements would not necessarily influence global organization. Second, altered global topology is more likely a trait rather than state, since disrupted regional centrality in our finding largely overlap with environmental^35^ and gene-environment risk to MDD^40^. Third, the global topology have a non-linear relationship with illness severity, or simply that disrupted connectome may not result in more severe depression symptom. Lastly, the global topology may be mixture of trait and state, however the effect size of global network difference is rather small so we did not detect difference with current power.

Several limitations restrict interpretability of our study. The cross-sectional design prevented us from investigating causality, the relevant precursor and outcomes. Second, the SCN approach produces all measurements at the group level, and the methodology inherently prevents us from exploring association with illness features, thus we cannot delineate the contribution of disturbance in one particular illness dimension. Third, the definition of node for network construction is still an open question. As in our study, applying different parcellation schemes yields variations in nodal properties.

In summary, we have shown that a cortical thickness coupling network shifted toward randomness, driven by a less clustered organization. Local hubs in depressed brains were primarily distributed in para-limbic cortices, which are well established abnormal regions in MDD. Importantly, disrupted nodal centralities formed the core of intrinsic large-scale networks, namely the default mode, salience and executive networks. Depression severity is associated with a subnetwork of these three large-scale networks. Current results from SCN highlight the underlying neuropsychological mechanism in MDD.

## Methods

### Participants and imaging protocol

Initially, 124 healthy control (HC) participants and 82 consecutively recruited MDD outpatients were included. Study participants in both groups were adults (age range, 18–60 years), right handed, and matched for sex and education levels. They underwent a diagnostic interview by experienced doctors using the Structured Clinical Interview for Diagnostic and Statistical Manual of Mental Disorders, 4th edition for Axis I Disorders. The aim was to investigate a relatively homogenous depression sample; therefore, the following criteria were used to exclude common confounders: i. historically/currently undergoing any course of anti-depression therapy (antidepressant, psychotherapy); ii. history of manic, hypomanic and/ or psychotic symptoms; iii. comorbidity of other Axis-I disorders; iv. current major medical and/or neurological conditions; v. history of head injury and/or substances abuse; and vi. contradictions to MRI scanning. Depression severity was rated using the 17-item Hamilton Depression Rating Scale (17-HDRS) by interview, as well as self-report scales in the Beck Depression Inventory-II (BDI-II). The study was performed in accordance with the Helsinki Declaration, and approved by the Institutional Review Board of Chongqing Medical University for the protection of human subjects. Written informed consent was obtained from each participant.

All images were acquired on a 3.0 tesla Siemens Trio MRI scanner using a 12-channel whole-brain coil (Siemens Medical, Erlangen, Germany). High-resolution T1-weighted structural images were acquired by magnetisation-prepared rapid gradient echo sequence (echo time = 2.52 ms; repetition time = 1900 ms; inversion time = 900 ms; flip angle = 9°; slices = 176; field of view = 256 × 256; voxel size = 1×1×1 mm^3^). All images underwent rigorous quality checks by a neurologist and radiologist. Eight controls were excluded from further analysis (7 for excessive head-motion and 1 for globus pallidus calcification) and 6 patients were excluded (4 for excessive head-motion, 1 for asymptomatic cerebral infarction and 1 for asymptomatic cerebral atrophy), therefore 76 patients and 116 controls were included in the final analysis.

### Cortical surface reconstruction and measures

Surface-based cortical reconstruction was performed in FreeSurfer (Version 5.3, https://surfer.nmr.mgh.harvard.edu). In brief, T1 images were corrected for intensity non-uniformities, with skull stripping performed. In each hemisphere, the white matter was segmented and the surface generated by tessellation. After correcting for topological defects, the pial surface was produced by nudging the white surface outwards. Cortical thickness was measured by calculating the shortest distance from the white matter to the pial surface at each vortex. During the reconstruction, several check-points (skull strip, white matter segments and pial surface) were visually inspected and major errors were manually corrected. Because of the semi-automatic nature of processing, group labels were concealed from the analysers to avoid bias.

### Network construction

Cortical thickness was extracted from 34 regions of interest (ROIs) in each hemisphere, defined using the Desikan–Killiany Atlas^62^. This gyral-based anatomical atlas is highly valid and reliable. These ROIs were defined as the network nodes, and linear correlation between two ROIs across subjects defined as edges. Each ROI was fitted using a linear regression model to remove the effects of age, sex (and their interactions), educated years and average hemisphere cortical thickness. Residuals were substituted as the net thickness to calculate Pearson–Correlation coefficients for all ROI pairs (i and j). Partial correlations were not chosen to define edges because one study reported underestimated topological measures^63^.

**Figure 5:**
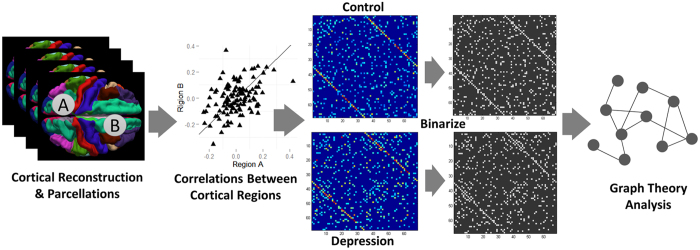
Schematic for construction of cortical thickness covariance networks and analysis workflow (left to right). The whole cerebral cortex was parcellated into 34 ROIs per hemisphere. Thickness was calculated after removing nuisance variables, and then correlation of all possible thickness pairs computed (total of 2278), and displayed as a correlation graph (minimal density of two fully connected graphs with weak correlations removed). Graphs were thresholded at a set density (0.12–0.4, 0.02 interval) for graph-theoretical analysis.

The 68 × 68 correlation matrices M_ij_ obtained in each group were thresholded to create a sparse graph. First, self and negative connections were set to 0, and the element c_ij_ in matrices above certain thresholds set to 1 (or otherwise 0). The minimal density of fully connected networks was 11.85%. Because the proportion of connected nodes in a network has a fundamental influence on topological measures, all metrics were explored as a function of connection density (density range of 0.12–0.4, with 0.02 intervals; Fig. 5). In addition, all substantial covariance connections that survived from false discovery rate (FDR) correction were identified. All negative connections were excluded because negative edges are difficult to interpret. Fisher’s r-to-z transformation was applied to obtain between-group differences for valid connections (Details in the Supplemental Material). Group differences were transformed into r for visualization (Fig. S4). For validation, an additional analysis was performed using 148 ROIs obtained by Destrieux parcellation^64^ (Details in the Supplemental Material).

### Topological analysis

Graph theory provides a theoretical framework for identifying the collective behaviour of a network. At the macro-scale, the connectome is organized neither regularly nor completely randomly, but exhibits an equilibrium state between these two extremes. The human brain connectome generally exhibits small-world architecture. Small-worldness (σ) is scalar and characterized by two critical metrics: a clustering coefficient (C), which measures segregation and is defined as a fraction of the node’s neighbours that are neighbours of each other; and characteristic path length (L), which measures integration and denotes the average shortest path length between all pairs of nodes in the network. Usually C and L are compared against randomly generated null networks for normalization, thus γ = C_real_/C_null_, λ = L_real_/L_null_ and σ = γ/λ. If σ >> 1, the network is considered to be “small-world”^65^. Twenty null networks (preserved for equal number of edges, degrees and same degree distribution of constructed real network) were generated as benchmarks to acquire small-worldness. Small-world behaviour can also be quantified by a network’s capacity to exchange information efficiently. The efficiency (E) metrics, E_global_ and E_local_, are similar to L and C but bear distinct physical meanings^17^. The cerebral connectome gives rise to hub nodes, which facilitate information flow in a network. The fact that a node becomes a hub might be because of its abundant connections (degree centrality, defined as the number of edge in a node) to another node. It might also indicate a low degree, which lies in the middle of a shortcut to other nodes, that is betweenness centrality (BC), defined as the fraction of all shortest paths in the network that pass through a given node. Information flow through a high BC node indicates high transmission efficiency. Both degree and BC were used to identify hubs. A node is considered to be a hub if either its degree or BC is one standard deviation above the average across nodes.

Association between illness severity and topology were investigated by dividing patients into “less severe” and “more severe” subgroups based on principal components analysis of HAMD-17 and BDI-II scores. The method was applied in previous literature^66^, and current analysis was performed by package “psych” in R. This procedure construct independent new variables (principal components) of which could best characterize the original data; as result the first principal component score explained 82% variance of HAMD-17 and BDI-II scores. Negative scores reflect less severe depressive symptoms (n = 40), and vice versa (n = 36). The demographic features as well as all network metrics were compared using the same strategy as above.

### Topology statistics between groups

The across-subjects covariance approach produces only one graph for each group at each sparsity level. For null-hypothesis testing of each metric, a bootstrap was applied to generate p-values and confidence intervals. This method randomly assigns subjects into pseudo control or patient groups iteratively with a fixed original group size. Correlation matrices were constructed and thresholded into a binary graph, then topological metrics computed as described above. At each sparsity, original topological metric differences between healthy and depressed groups were tested against the bootstrap null network (α = 0.05, two-tailed, 5000 permutations). Because topological metrics are sensitive to network density thresholds, additional analyses, specifically, area under the curve (AUC) analysis and functional data analysis (FDA), were performed on both global and regional metrics across all network density thresholds. The AUC analysis depicts the curves of topological measures changes varied as function of densities. FDA analysis overcome the shortcoming in AUC, that is AUC might be too sensitive when network density is high and is further insensitive to differences in the shape of the curves rather their mean. The implemented methods were described in GAT toolbox in detail^67^. Using these analyses, group comparisons are more robust to the thresholding procedure in network analysis. Comparing sparsity-metric curves against null also utilizes permutation procedures. Inflation of Type-I errors by multiple comparisons were controlled by FDR correction procedures. Graph Analysis Toolbox^67^ and Brain Connectivity Toolbox^68^ were used to implement the analyses. Connectome figures were visualized using the BrainNet Viewer^69^ (www.nitrc.org/projects/bnv/).

## Additional Information

**How to cite this article**: Wang, T. *et al*. Disorganized cortical thickness covariance network in major depressive disorder implicated by aberrant hubs in large-scale networks. *Sci. Rep.* **6**, 27964; doi: 10.1038/srep27964 (2016).

## Supplementary Material

Supplementary Information

## Figures and Tables

**Table 1:** Clinical features of healthy control, depression patient, and patient subgroup.

	HC (n = 116)	MDD (n = 76)	Statistics
Female: Male	72:44	48:28	p = 1, Χ^2^ = 0#
Age (Year)	35.3 ± 12.1	36.2 ± 11.3	t = −0.53, p = 0.60^+^
Years of Education	12.8 ± 4.0	12.1 ± 3.6	t = 1.29, p = 0.20^+^
Age of Onset (Year)	NA	33.8 ± 1.3	NA
HDRS-17 score	1.9 ± 1.9	22.7 ± 4.6	t = −37.5, p < 2.2e-16^+^
BDI-II score	4.9 ± 5.5	22.6 ± 7.8	t = −17.2, p < 2.2e-16^+^
Subgroup of MDD	Low Severity	High Severity	
Female: Male	24:16	24:12	Χ^2^ = 0.13#, p = 0.72
Age (Year)	35.72 ± 11.02	36.69 ± 11.65	t = 0.37, p = 0.71^+^
Years of Education	12.07 ± 3.68	12.11 ± 3.55	t = 0.04, p = 0.97^+^
Age of Onset (Year)	34.35 ± 11.21	35.53 ± 11.74	t = 0.45, p = 0.66^+^
HDRS-17 score	20.05 ± 3.68	25.58 ± 4.16	t = 6.49, p < 1.38 e-8^+^
BDI-II score	17.38 ± 5.81	28.42 ± 5.15	t = 8.78, p < 4.27 e-13^+^

^#^Chi-square test. ^+^Student’s *t*-test, two-tailed. Numerical data are shown as mean ± SD.

**Table 2:** Nodal topological differences between HC and MDD groups.

Node & Hub Affiliation#	Brodmann Area & Network^§^	Direction	*p*-value (FDR corrected)
Betweenness Centrality			
L- Inferior parietal (MDD)	BA 39, DMN	MDD > HC	0.015
L- isthmus cingulate (MDD)	BA 30, DMN	MDD > HC	0.012
R- Inferior parietal (MDD)	BA 39, CEN	MDD > HC	0.016
R- isthmus cingulate (MDD)	BA 30, DMN	MDD > HC	0.050
R- supra-marginal (MDD)	BA 40, CEN	MDD > HC	0.041
R- temporal pole (MDD)	BA 38, SN	MDD > HC	0.034
L- mid-temporal (HC)	BA 31, DMN	HC > MDD	0.047
L- pars opercularis (non-hub)	BA 44, SN	HC > MDD	0.022
L- supra-marginal (HC)	BA 40, CEN	HC > MDD	0.047
R- caudal middle frontal (HC)	BA 6, CEN	HC > MDD	0.033
R- transverse temporal (HC)	BA 41 & 42, N/A	HC > MDD	0.002
R- insula (HC)	N/A, SN	HC > MDD	0.023
Degree Centrality			
L- lateral occipital (non-hub)	BA 17, 18 & 19, N/A	MDD > HC	0.022
L- isthmus cingulate (MDD)	BA 30, DMN	MDD > HC	0.041
L- mid temporal (HC)	BA 31, DMN	HC > MDD	0.034
L- frontal pole (non-hub)	BA 10, N/A	HC > MDD	0.030
R- transverse temporal (HC)	BA 41 & 42, N/A	HC > MDD	0.038
R- insula (HC)	N/A, SN	HC > MDD	0.001
Efficiency			
L- rostral middle frontal	BA 9, CEN	HC > MDD	0.046
R- caudal anterior cingulate	BA 24, SN	MDD > HC	0.041
R- cuneus	BA 17, N/A	MDD > HC	0.027
R- entorhinal	BA 28 & 34, N/A	HC > MDD	0.018
R- temporal pole	BA 38, SN	HC > MDD	0.017

^#^Hub Affiliation indicate whether the node is hub in either group. ^**§**^Gyral-based parcellation was approximately assigned to Brodmann areas, and components of large scale network (i.e default mode network/ DMN, central executive network/ CEN, salience network/SN).

**Table 3:** Altered nodal topology associated with depression severity in patient group.

Node	Brodmann Area#	Direction	p-value (FDR corrected)
Betweenness Centrality			
L- Inferior parietal	BA 39, CEN	High < Low	0.003
L- lateral orbitofrontal	BA 11, N/A	High < Low	0.005
L- posterior cingulate	BA 23 & 24, DMN	High > Low	0.048
R- Inferior parietal	BA 39, CEN	High > Low	0.028
R- posterior cingulate	BA 23 & 24, DMN	High > Low	0.022
Degree Centrality			
L- medial orbitofrontal	BA 11 & 25, DMN	High > Low	0.025
R- superior parietal	BA 5 & 7, CEN	High > Low	0.022
R- superior temporal	BA 41 & 42, N/A	High < Low	0.041
Efficiency			
L- cuneus	BA 17, N/A	High > Low	0.045
L- pars opercularis frontal	BA 44, CEN	High < Low	0.046
L- rostral middle frontal	BA 9, CEN	High < Low	0.008
R- middle temporal	BA 31, DMN	High > Low	0.024

^#^Gyral-based parcellation was approximately assigned to Brodmann areas, and components of large scale network.
