# Supplementary material for: Disorganized cortical thickness covariance network in major depressive disorder implicated by aberrant hubs in large-scale networks
Source: Sci Rep. 2016 Jun 15;6:27964. doi: 10.1038/srep27964 (PMC4908416; doi:10.1038/srep27964)
Supplement: Supplementary Information [file srep27964-s1.pdf]

Supplemental Material

Disorganized cortical thickness covariance network in major depressive disorder implicated by aberrant hubs in a large-scale network

Tao Wang, Kangcheng Wang , Hang Qu, Jingjing Zhou, Qi Li, Zhou Deng, Xue Du , Fajing Lv, Gaoping Ren, Jing Guo, Jiang Qiu, Peng Xie

Content:

1. Differences of structural covariance connectivity between two group.
2. Validation in 148*148 graph analysis.
    - 2.1 Network Construction
    - 2.2 Differences in Global Topology
    - 2.3 Identification of Hubs
    - 2.4 Differences in Centrality

1. Difference of each pair of structural covariance connectivity between two group

To demonstrate altered pairwise covariance network, We first excluded all negative connections since negative edge is difficult to interpret. Pearson-correlation between two regions were computed, and statistical significant pair was defined as substantial covariance connectivity. A network contains 50 nodes and 228 connections survived FDR-correction. Fisher’s r-to-z transformation were applied to acquire between group difference. The difference was transformed into correlation coefficient (Pearson r) for visualization in Figure S1. To summarize, we identified a total of 64 pairs of structural connectivity in MDD differed from HC. The overall changes in structural connectivity are dominated by disconnection, which 44 decreasing and 20 increasing connectivity are found. Of the 44 pairs of decreased connectivity, twenty-one pairs were intra-hemisphere (9 in left and 12 in right), 17 pairs were inter-hemisphere and 6 were homotopic connections.

Figure S1. Differences of SCN between HC and MDD. Red color indicate greater connection strength in MDD, blue indicate lower connection strength in MDD, line-width indicate the magnitude of change.

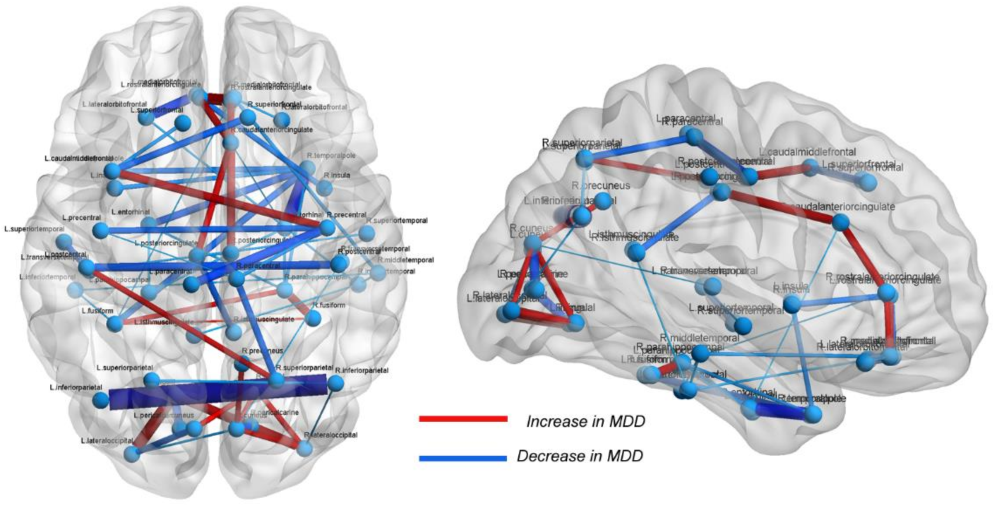

2. Validation in 148*148 graph analysis

2.1 Network Construction

Parcellation schemes have an effects on topological measurements (Fornito, Zalesky, & Bullmore, 2010; Wang et al., 2009; Zalesky et al., 2010). We performed additional network analysis for validation using same analysis strategy in manuscript. Each hemisphere was divided into 74 ROIs (Destrieux, Fischl, Dale, & Halgren, 2010) and computed thickness, confounders (age, sex, age-sex interaction, education year, overall thickness) were removed by linear regression. Pearson-correlation coefficient were calculated between each pair of thickness' residual, resulted 148*148 graph. Self-connection in diagonal elements and negative connections were removed. The minimal density of which 2 networks fully connected was 4.66% (Figure S2).

Figure S2. Thresholed 148*148 correlation graph at minimal density (4.66%).

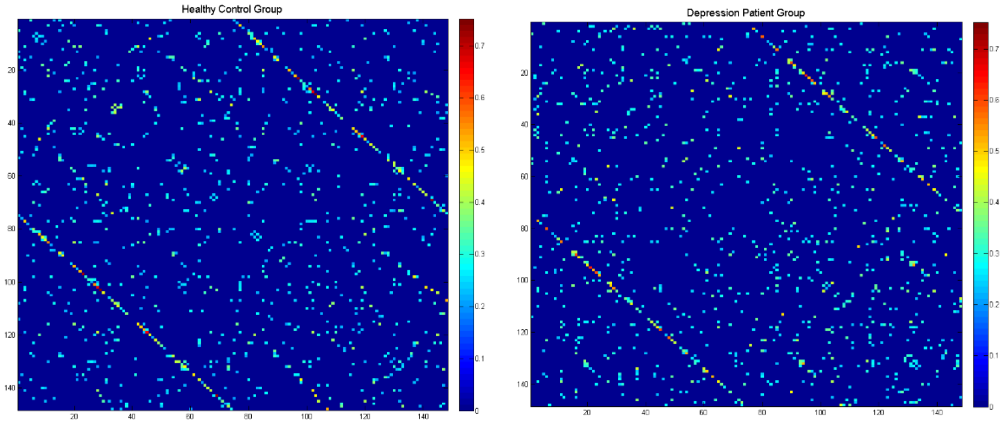

Correlation Graph of Healthy Controls | Correlation Graph of Depression Patients

2.2 Differences in Global Topology

We filtered weak connections in range of connection density (5% ~ 35%, 2% incremental in each step) to derive binary network for analysis. Twenty null networks (preserved degree distribution) were generated as benchmark network for topological comparison. For hypothesis testing, 1000 bootstrap samples were generated as null, p-values and confidence intervals were calculate against null.

Result shows that constructed network still preserves small-world property. In addition, alteration of global topology still hold compared to 68*68 networks. Small-World, gamma (normalized clustering coefficient) and local efficiency are lower in patient group (Table S1, Figure S3).

Tables S1. Alteration in global topology between HC and MDD. Significant lower small-world (Sigma) are driven by lower gamma/ Efficiency$_{local}$

Global Metrics	Differences Direction	AUC/ FDA p-values (FDR)
**Characteristic Path Length**	MDD < HC	0.149 / 0.162
**Lambda (norm L)**	MDD < HC	0.122 / 0.143
**Global Efficiency**	MDD < HC	0.135 / 0.152
**Clustering Coefficient**	MDD < HC	0.062 / 0.060
**Gamma (norm C)**	MDD < HC	0.006 / 0.004
**Local Efficiency**	MDD < HC	0.013 / 0.012
**Sigma**	MDD < HC	0.005 / 0.002

Figure S3. Small-World measurements as function of connection density

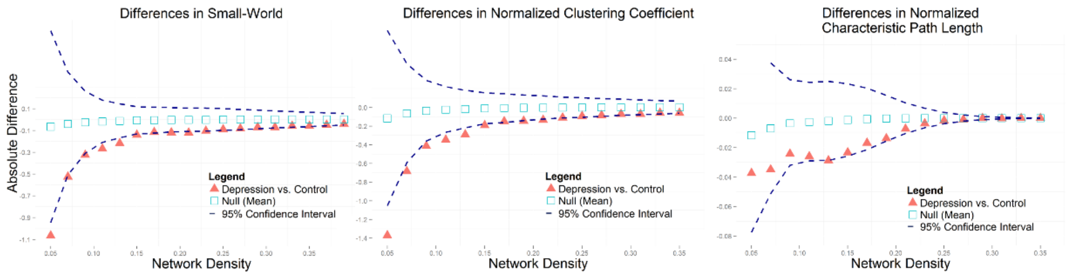

Left to Right: Difference in Small-world, Gamma (normalized clustering coefficient) and Lambda (normalized characteristic path length)

2.3 Identification of Hubs

Figure S4. We identified hubs by criteria of above 1.5 standard deviation of all nodal centrality.

Hubs in Healthy Controls

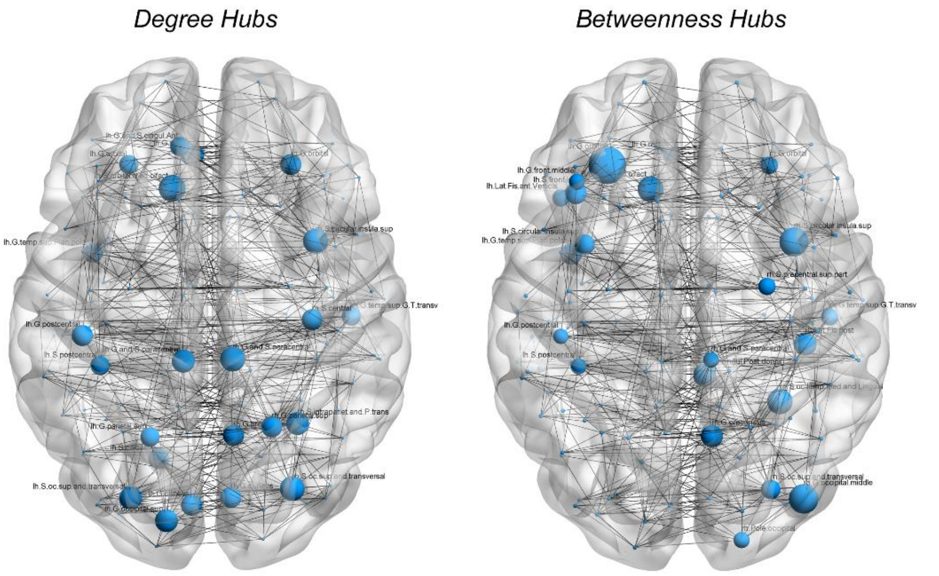

Hubs in Depression Patients

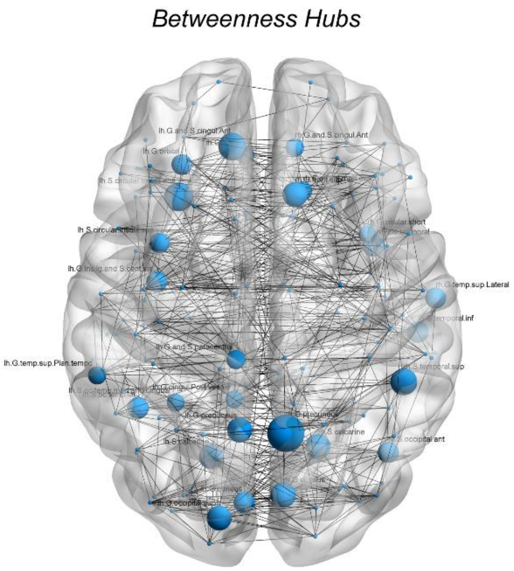

2.4 Difference in centrality

Figure S5. Difference in centrality measures across densities (5% - 35%, 5% incremental) were localized by AUC and FDA procedure (1000 permutations.).

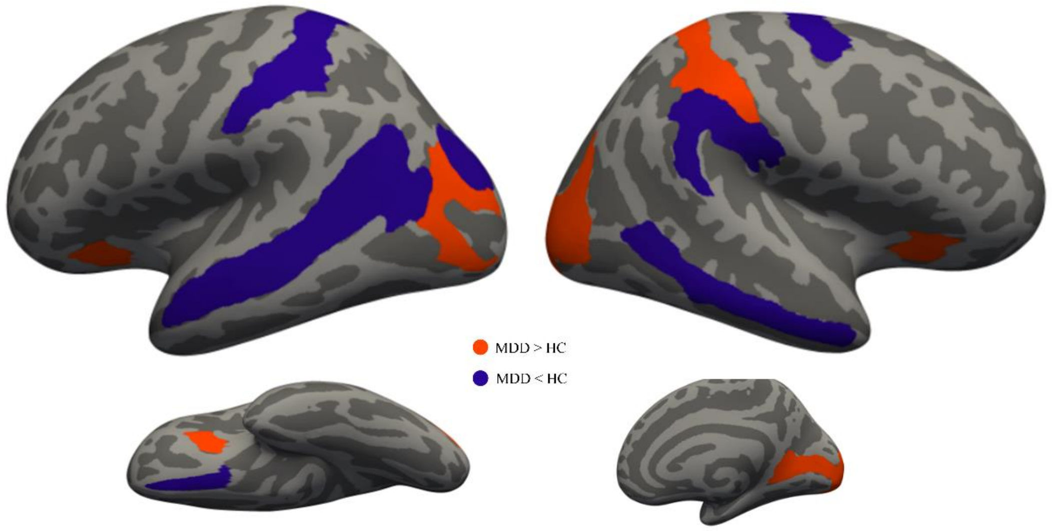

Table S2. Regions with altered nodal centrality between groups

**Nodal Degree**	**Direction**	**AUC/ FDA p-values (FDR)**	
lh.G.occipital.middle	MDD > HC	0.003	0.003
lh.S.circular.insula.ant	MDD > HC	0.029	0.023
lh.S.orbital.med.olfact	MDD < HC	0.021	0.021
lh.S.postcentral	MDD < HC	0.03	0.025
lh.S.temporal.sup	MDD < HC	0.038	0.041
rh.G.occipital.middle	MDD > HC	0.031	0.037
Rh.Pole.occipital	MDD > HC	0.043	0.038
Rh.S.calcarine	MDD > HC	0.023	0.021
rh.S.oc.middle.and.Lunatus	MDD > HC	0.027	0.023
rh.S.postcentral	MDD < HC	0.022	0.023
**Nodal Betweenness**			
lh.S.circular.insula.ant	MDD > HC	0.049	0.036
lh.S.oc.sup.and.transversal	MDD < HC	0.030	0.031
rh.G.pariet.inf.Supramar	MDD > HC	0.014	0.012
rh.G.temporal.middle	MDD < HC	0.006	0.015
rh.S.calcarine	MDD > HC	0.041	0.038
rh.S.precentral.sup.part	MDD < HC	0.04	0.031

References:

Destrieux, C., Fischl, B., Dale, A., & Halgren, E. (2010). Automatic parcellation of human cortical gyri and sulci using standard anatomical nomenclature. *Neuroimage, 53*(1), 1-15. doi:10.1016/j.neuroimage.2010.06.010

Fornito, A., Zalesky, A., & Bullmore, E. T. (2010). Network scaling effects in graph analytic studies of human resting-state FMRI data. *Front Syst Neurosci, 4*, 22. doi:10.3389/fnsys.2010.00022

Wang, J., Wang, L., Zang, Y., Yang, H., Tang, H., Gong, Q., . . . He, Y. (2009). Parcellation-dependent small-world brain functional networks: a resting-state fMRI study. *Hum Brain Mapp, 30*(5), 1511-1523. doi:10.1002/hbm.20623

Zalesky, A., Fornito, A., Harding, I. H., Cocchi, L., Yucel, M., Pantelis, C., & Bullmore, E. T. (2010). Whole-brain anatomical networks: does the choice of nodes matter? *Neuroimage, 50*(3), 970-983. doi:10.1016/j.neuroimage.2009.12.027
